# The Development of a Non-Invasive Screening Method Based on Serum microRNAs to Quantify the Percentage of Liver Steatosis

**DOI:** 10.3390/biom14111423

**Published:** 2024-11-08

**Authors:** Polina Soluyanova, Guillermo Quintás, Álvaro Pérez-Rubio, Iván Rienda, Erika Moro, Marcel van Herwijnen, Marcha Verheijen, Florian Caiment, Judith Pérez-Rojas, Ramón Trullenque-Juan, Eugenia Pareja, Ramiro Jover

**Affiliations:** 1 Unidad Mixta de Investigación en Hepatología Experimental, IIS Hospital La Fe, 46026 Valencia, Spain; polina_soluyanova@externos.iislafe.es (P.S.); erika_moro@externos.iislafe.es (E.M.); 2 Departamento de Bioquímica y Biología Molecular, Universidad de Valencia, 46010 Valencia, Spain; 3 Health and Biomedicine, LEITAT Technological Center, 08225 Terrassa, Spain; gquintas@leitat.org; 4 Centro de Investigación Biomédica en Red de Enfermedades Hepáticas y Digestivas (CIBERehd), ISCIII, 28029 Madrid, Spain; 5 Servicio de Cirugía General y Aparato Digestivo, Hospital Universitario Dr. Peset, 46017 Valencia, Spain; alvaroprubio@gmail.com (Á.P.-R.); pareja_eug@gva.es (E.P.); 6 Pathology Department, Hospital Universitario y Politécnico La Fe, 46026 Valencia, Spain; rienda_iva@gva.es (I.R.); perez_jud@gva.es (J.P.-R.); 7 Department of Translational Genomics, Research Institute of Oncology and Developmental Biology (GROW), Maastricht University, 6229-ER Maastricht, The Netherlands; m.vanherwijnen@maastrichtuniversity.nl (M.v.H.); marcha.verheijen@maastrichtuniversity.nl (M.V.); florian.caiment@maastrichtuniversity.nl (F.C.)

**Keywords:** Metabolic dysfunction-associated steatotic liver disease, microRNA, serum biomarker, liver steatosis, quantitative prediction, partial least squares regression

## Abstract

Metabolic dysfunction-associated steatotic liver disease (MASLD) is often asymptomatic and underdiagnosed; consequently, there is a demand for simple, non-invasive diagnostic tools. In this study, we developed a method to quantify liver steatosis based on miRNAs, present in liver and serum, that correlate with liver fat. The miRNAs were analyzed by miRNAseq in liver samples from two cohorts of patients with a precise quantification of liver steatosis. Common miRNAs showing correlation with liver steatosis were validated by RT-qPCR in paired liver and serum samples. Multivariate models were built using partial least squares (PLS) regression to predict the percentage of liver steatosis from serum miRNA levels. Leave-one-out cross validation and external validation were used for model selection and to estimate predictive performance. The miRNAseq results disclosed (a) 144 miRNAs correlating with triglycerides in a set of liver biobank samples (*n* = 20); and (b) 124 and 102 miRNAs correlating with steatosis by biopsy digital image and MRI analyses, respectively, in liver samples from morbidly obese patients (*n* = 24). However, only 35 miRNAs were common in both sets of samples. RT-qPCR allowed to validate the correlation of 10 miRNAs in paired liver and serum samples. The development of PLS models to quantitatively predict steatosis demonstrated that the combination of serum miR-145-3p, 122-5p, 143-3p, 500a-5p, and 182-5p provided the lowest root mean square error of cross validation (RMSECV = 1.1, *p*-value = 0.005). External validation of this model with a cohort of mixed MASLD patients (*n* = 25) showed a root mean squared error of prediction (RMSEP) of 5.3. In conclusion, it is possible to predict the percentage of hepatic steatosis with a low error rate by quantifying the serum level of five miRNAs using a cost-effective and easy-to-implement RT-qPCR method.

## 1. Introduction

Metabolic dysfunction-associated steatotic liver disease (MASLD), previously referred to as nonalcoholic fatty liver disease (NAFLD), affects approximately 30% of adults globally and is the most common cause of chronic liver disease and the leading cause of liver-related morbidity and mortality worldwide [1,2]. 

Hepatocellular steatosis is the hallmark of MASLD. The histological criterion for the diagnosis of MASLD is the presence of fat droplets in more than 5% of the hepatocytes.

Numerous intra- and extra-hepatic mechanisms, including adipose tissue dysfunction, altered gut microbiota, and genetic pre-disposition are recognized drivers for MASLD development. Hepatocellular lipid accumulation, the basis of this disease, results from an imbalance between synthesis and utilization of lipids. Dysregulation of lipid homeostasis in hepatocytes leads to lipotoxicity that result in dysfunctional organelles (endoplasmic reticulum stress, mitochondrial/lysosomal dysfunction, impaired autophagy, etc.) promoting inflammation, hepatocellular damage, and cell death [3].

Given the high prevalence of MASLD, having a rapid, easy, and non-invasive screening method for the general population would be highly recommended.

Currently, liver biopsy is the gold standard for diagnosing and staging steatosis, but its application is limited by the invasive nature, risk of complications, high cost, and poor acceptance by patients. Moreover, there are significant inter-observer variability and sampling errors. Furthermore, the applicability of liver biopsy to the very large population affected by MASLD is not feasible.

Various imaging modalities have also been developed for non-invasively detecting the presence of, or quantifying, liver steatosis. Ultrasonography is a well-established and lower-cost imaging technique for steatosis, yet it is limited by its low sensitivity for low-mild steatosis and for being highly operator-dependent. Computed tomography presents a potential radiation hazard. Moreover, it also lacks the sensitivity to detect mild steatosis and small changes in fat content. A controlled attenuation parameter based on the FibroScan^®^ technology is a promising tool for a semiquantitative assessment of steatosis, but the accuracy rate depends on the operator’s expertise and is affected by age, width of the intercostal space, skin capsular distance, and body mass index. Magnetic resonance imaging (MRI) is regarded as the most accurate quantitative method for steatosis in clinical practice, especially for longitudinal follow up. However, the MRI scanners, in contrast to ultrasonographs, are costly, resource-demanding, and frequently not applicable to large populations and to specific patients (young children, claustrophobia, implanted electronic devices, metal implants, etc.) [4,5,6].

Therefore, there is a demand for non-invasive methods, easily applicable to the general population as screening or surveillance methods, that allow incipient steatosis to be detected with confidence. MASLD is often described as a silent disease, in which many patients are diagnosed incidentally [7]. Consequently, underdiagnosis is common, particularly in mild cases, attributed to its asymptomatic nature and the traditional limited sensitivity of imaging techniques. A high throughput screening method, applicable to serum, for the assessment of hepatic steatosis in the population would possibly be the best ally to apply preventive medicine to the continuously growing MASLD pandemic.

Profound gene expression reprogramming, including altered miRNA expression, is parallel to the pathophysiological mechanisms driving MASLD. Indeed, changes in hepatic miRNA expression patterns are associated with MASLD at early, intermediate, and late stages, and specific miRNA species appear to be involved in steatosis development and in the progression of this increasingly prevalent disease [8].

Consequently, several studies aimed at identifying circulating biomarkers for MASLD have focused on miRNAs. Surprisingly, the miRNAs found in these independent studies show a very limited overlap [9]. Such a lack of agreement is likely to come from the bias in miRNA pre-selection, the reduced number of patients included, different technical approaches to quantify them, no biopsy-proven diagnosis, etc. Moreover, most of these studies focused on MASLD severity prediction, and none of them aimed to be a quantitative method for steatosis [9].

In a recent pilot study, we attempted to use serum miRNAs as a quantitative approach to predict the percentage of liver steatosis. However, the conclusions of this study based solely on miRNAseq data were limited by a low sample size, lack of validation with other methods and patient cohorts, and because serum and liver samples were not paired from the same MASLD patients [10]. In the present study, we have extended and reinforced this pilot study and robustly shown that there are a small group of liver miRNAs that correlate with the liver lipid content in MASLD patients from different cohorts. These liver miRNAs are also detected in the serum of the same patients, where they preserve correlation with liver fat content. After the development of a regression model, externally validated, we conclude it is possible to predict the percentage of liver steatosis with a low error rate by quantifying a small subset of serum miRNAs using an easily implementable and cost-effective RT-qPCR method.

## 2. Materials and Methods

### 2.1. Patients and Human Samples

Two different cohorts of patients were chosen to identify miRNAs correlating with the amount of liver fat (% of steatosis).

The first cohort was already described in our previous pilot study [10] and consisted of 20 organ donors who were primarily assigned to liver transplantation, but their livers failed to be transplanted and were donated to research (Biobank Hospital La Fe, Valencia, Spain). These livers showed a wide range of steatosis. The concentration of triglycerides (TGs) was determined in these liver samples by biochemical analyses. Liver RNA isolation and miRNAseq were performed [10]. Sequencing results were deposited in ENA under accession PRJEB53387.

The second cohort included 39 morbidly obese patients recruited between 2020 and 2023 and referred to the Esophageal-Gastric Surgery Section of the Dr. Peset University Hospital in Valencia. Twenty-four of these patients were for miRNA discovery and model development, and 15 for external validation. All the patients fulfilled criteria for bariatric surgery (BS) according to the 1991 NIH guideline for eligibility [11]. Exclusion criteria included obesity secondary to endocrinopathies, active neoplastic disease, severe psychiatric disorder, alcoholism or drug dependence, non-adherence to recommended hygienic-dietary changes, anesthetic contraindication, and severe comorbidities.

On the day of surgery, a scheduled wedge liver biopsy was taken. Hematoxylin-eosin (HE) and Masson’s trichrome-stained paraffin-embedded liver biopsy sections were examined and interpreted by the same experienced hepatopathologists, who were unaware of the patients’ clinical data. Steatosis, along with ballooning, lobular inflammation, and fibrosis, were assessed as outlined by Brunt et al. [12]. Disease activity was scored according to SAF [13].

HE-stained biopsy sections were digitalized to automatically estimate the % of steatosis. Whole slide images (WSIs) were captured using the Aperio CS2 scanner (Leica Biosystems, Nussloch, Germany) at 20× (equivalent to 0.50 µm/pixel) in SVS format (Apeiro ImageScope 12.3.3.5048, Leica Biosystems, Vista, CA, USA). The % of steatosis in each sample was quantified using a multivariate model based on nine morphological features (eccentricity, area, circularity, perimeter, equivalent diameter, extent, major axis length, minor axis length, and solidity) of each object found in the WSI after image pre-processing and segmentation [14]. The % of steatosis in each WSI was estimated as 100 × (area steatotic objects)/(area tissue + area steatotic objects + area non-steatotic objects).

The % of liver steatosis was also assessed by magnetic resonance imaging (MRI)-proton density fat fraction (PDFF) (mDixonQuant, Philips Healthcare, Best, The Netherlands).

Venous blood was extracted after overnight fasting, by the time of liver biopsy. Blood was collected in siliconized tubes, allowed to clot, and centrifuged at 2500× *g* for 10 min. Serum samples were stored at −80 °C.

For model development, we selected 24 morbidly obese patients, whose baseline characteristics are presented in Table 1A. For external model validation, we selected 15 morbidly obese patients + 10 non-morbidly obese patients, with biopsy proven MASLD (previously described in [9]), whose baseline characteristics are presented in Table 1B,C, respectively. In general, patients showed non-severe MASLD with disease activities ≤ 2 and no or very mild fibrosis (Table 1A–C).

Informed written consent was obtained from all patients, and the study was conducted in conformity with the Helsinki Declaration and was approved by the Human Ethics Committees of La Fe University Hospital (n° 2013/0232, 2012/0452 and 2022-204-1) and Dr. Peset University Hospital (n° 44/19) in Valencia, Spain.

### 2.2. RNA Isolation, Small RNA Library Preparation and miRNAseq Analysis

The total RNA was isolated from liver samples using the miRNeasy mini kit (Qiagen Westburg BV, Leusden, The Netherlands) according to the manufacturer’s protocol, followed by a DNAse I treatment (Qiagen Inc., Venlo, The Netherlands). RNA concentration and quality were measured using a BioAnalyzer system (Agilent Technologies, Breda, The Netherlands). Starting from total RNA samples, small RNAs were size selected and ligated for sequencing following the NEXTFLEX Small RNA-Seq Kit v3 (NOVA-5132-06, Perkin Elmer, Waltham, MA, USA). TruSeq Small Prep Kit Preparation (15004197 Rev. D, Illumina, Eindhoven, The Netherlands). Samples were sequenced on the HiSeq 6000 (Illumina) in single-end 100 bp. The Fastq files have been processed into miRNA count using the miRge3.0 v0.0.1 analysis software [15], using the default parameters. Normalization to reads per million (RPM) was performed. Sequencing data have been deposited on BioStudies under the accession number S-ONTX26.

### 2.3. miRNA Expression Analysis by RT-qPCR

The total RNA was extracted from liver tissue with the miRNeasy Mini Kit (Qiagen, Madrid, Spain) or from 300 μL of human serum using Trizol LS reagent (Invitrogen, Barcelona, Spain) followed by the miRNeasy Mini Kit. Prior to serum RNA purification, we added 20 μg of RNase-free glycogen (Roche Applied Sciences, Barcelona, Spain) as a carrier. Purified RNA (1 μg) was reverse transcribed in two steps: polyadenylation with 1U Poly(A) polymerase from *E. coli* (New England BioLabs, Ipswich, MA, USA) and reverse transcription with a universal anchor primer and 200U M-MLV reverse transcriptase (Invitrogen, Barcelona, Spain) as described in [16].

Diluted cDNA was amplified in a LightCycler 480 Instrument (Roche Applied Science, Barcelona, Spain) using LightCycler 480 Probes Master (Roche Applied Science) and the a universal reverse primer [16], a specific forward primer for each miRNA and a universal TaqMan probe (IDT, Leuven, Belgium) [16]. The concentration of miRNAs in the samples was calculated with the 2-ddCt method. Sample to sample variations were normalized with the geometric mean of two miRNAs: miR-15a-5p and miR-25-3p, which are abundantly expressed in human tissues and serum, and show low variability. These miRNAs showed the best stability scores of our datasets according to geNorm, an algorithm for evaluating and determining the most stable reference (housekeeping) genes from a gene expression dataset in a given sample panel [17]. These reference miRNAs have also been previously used in other studies as stable miRNAs for normalization.

Liver and serum sample datasets have been deposited on BioStudies under accession numbers S-ONTX27 and S-ONTX28, respectively.

### 2.4. Bioinformatic Analysis and Modeling

Feature selection in the miRNAseq datasets involved an initial univariate analysis of the linear association between the miRNA RPM and the concentration of liver TGs, or the % of steatosis by liver biopsy WSI digital analysis or by MRI-PDFF (mDixonQuant, Philips Healthcare, Best, the Netherlands), in two independent cohorts, using r ≥ 0.34 and *p* ≤ 0.1 as criteria for the identification of potentially relevant miRNAs. After RT-PCR validation in liver and serum of obese BS patients, the data set used for final model optimization comprised 10 miRNAs: miR-145-3p, miR-122-5p, miR-143-3p, miR-192-5p, miR-34a-5p, miR-500a-5p, miR-32-5p, miR-98-5p, miR-182-5p, and miR-93-5p. Multivariate models were built using partial least squares (PLS) regression to predict the hepatic steatosis level, estimated as the % of fat in biopsy images and by MRI-PDFF, based on serum miRNAs measured by RT-PCR. Leave-one-out cross validation (LOO-CV) was used for model selection and for the initial estimation of the model predictive performance. Furthermore, permutation testing (500 permutations) was used to assess the statistical significance of the root mean square error of cross validation (RMSECV), where the *p*-value was calculated as the fraction of the permuted models, providing better estimates than that observed for the original model. To increase the applicability of the approach, a feature selection step was included to reduce the number of miRNAs in the model down to 5. Accordingly, the performance of multivariate models built using each of the 252 possible combinations of 5 miRNAs among the list of 10 miRNAs in the data set, was estimated. Finally, the performance of the PLS model using the selected set of miRNAs was assessed by the prediction of an external validation set of 25 serum samples.

## 3. Results

### 3.1. Liver miRNAseq and Selection of miRNAs Showing Correlation with the Percentage of Steatosis

Two different subsets of liver samples were sequenced as follows: 20 from a human liver collection (Biobank Hospital La Fe, Valencia, Spain) covering a wide range of liver TGs; and 24 from a cohort of morbidly obese BS patients with variable steatosis as assessed by liver biopsy WSI quantification and by MRI-PDFF (Dixon). Starting from total liver RNA, small RNAs were size selected and quantified by miRNAseq. After data processing and normalization, the reads of each liver miRNA were associated with the % of steatosis by Pearson’s correlation.

**Figure 1:**
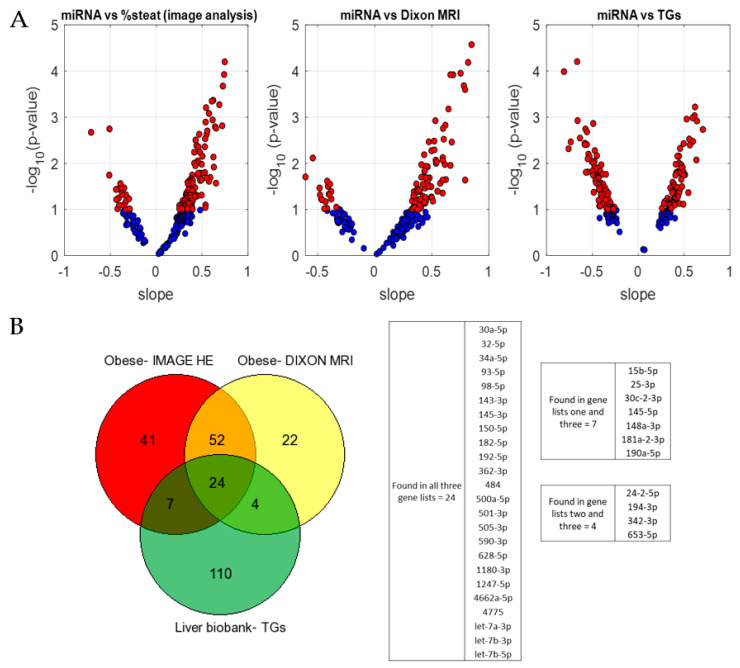
Identification of miRNAs showing correlation with the percentage of hepatic steatosis. Human liver samples from two cohorts of patients were analyzed by miRNAseq. The normalized number of reads was correlated with the % of liver steatosis as assessed by different linear methods. (**A**) Volcano plots showing miRNAs with an association of r ≥ 0.34 and *p*-value ≤ 0.1 as red dots. (**Left** and **middle** plots) liver samples from morbidly obese BS patients. (**Right** plot) liver samples from liver biobank cohort. (**B**) Venn diagram displaying the 35 miRNAs showing correlation with the % of steatosis in the two cohorts of patients.

For the initial selection of correlating miRNAs, we applied the following cut-offs: r ≥ 0.34 and *p*-value ≤ 0.1. With these settings we found the following: 144 miRNAs correlating with the concentration of liver TGs in the liver biobank sample cohort; and 124 and 102 miRNAs correlating with the % of steatosis by liver biopsy WSI and by MRI-PDFF analyses, respectively, in the morbidly obese cohort (Figure 1A and Supplementary Table S1).

Next, we reasoned that the miRNAs more likely associated with the liver lipid content would be those showing consistent correlation across the two independent cohorts of liver samples (biobank and morbidly obese patients). Using Venn diagrams, we observed that there were 24 miRNAs that showed correlation with steatosis in the two cohorts of patients, with the three steatosis quantification methods (Figure 1B). Moreover, we found 11 miRNAs that showed correlation with steatosis in the two cohorts of patients, but with only two of the three steatosis quantification methods (Figure 1B). Thus, 35 miRNAs were selected for further analyses. By identifying miRNAs that are consistently correlated with liver lipid content in both sets of liver samples, we aim to select miRNAs that are robust indicators of steatosis and have a reduced likelihood of false positives and negatives that may arise from cohort-specific variations and their associated factors.

### 3.2. Liver miRNA Validation by RTqPCR and Confirmation of Correlation with the Percentage of Steatosis

From the initial selection of 35 miRNAs, we attempted to validate 22 by RT-qPCR. We excluded 13 miRNAs for different reasons: due to technical limitations found during RT-qPCR assay development and performance, and/or because the miRNAs were low expression passenger strands. On the contrary, we included in the validation the liver specific miR-122-5p, which has a very high expression in human liver. The highest number of reads for miR-122-5p observed in the miRNAseq results could have led to its underestimation, further supporting its inclusion.

Results from the validation of the abovementioned set of 22 miRNAs and miR-122-5p in the cohort of 24 morbidly obese liver samples demonstrated that 18 of them (82%) confirmed correlation with liver steatosis (r ≥ 0.38 and *p*-value ≤ 0.1) (Table 2). However, miR-30a-5p, miR-1180-3p, miR-1247-5p, and let-7a-3p did not show association with liver lipid content based on data from RT-qPCR. Interestingly, miR-122-5p also showed correlation with steatosis when measured by RT-qPCR (Table 2).

In summary, using RT-qPCR, we confirmed the miRNAseq results showing association of 19 miRNAs with steatosis in the livers of the cohort of 24 morbidly obese patients. Next, we sought to determine if these miRNAs were detected in the sera of the same 24 patients and if these serum miRNAs preserved an association with the amount of liver fat.

### 3.3. Quantification of Selected miRNAs in Human Serum and Verification of Their Correlation with the Percentage of Steatosis

We attempted to measure, in serum, 19 miRNAs selected from the previous liver analyses. However, five of them (miR-15b-3p, miR-194-3p, miR-24-2-5p, miR-30c-2-3p, and let-7b-5p) showed technical limitations in the development and performance of RT-qPCR assays and were not further considered because of their unreliable quantification in serum.

Of the remaining 14 miRNAs reliably detected in serum, 10 of them (71%) showed association with hepatic steatosis, while four miRNAs were not validated (Table 3). Furthermore, among the list of 10 miRNAs, six of them showed association with liver steatosis independently of the method used for liver fat quantification.

Therefore, results demonstrate that the concentration of a small subset of serum miRNAs varies accordingly with the % of liver steatosis.

**Figure 2:**
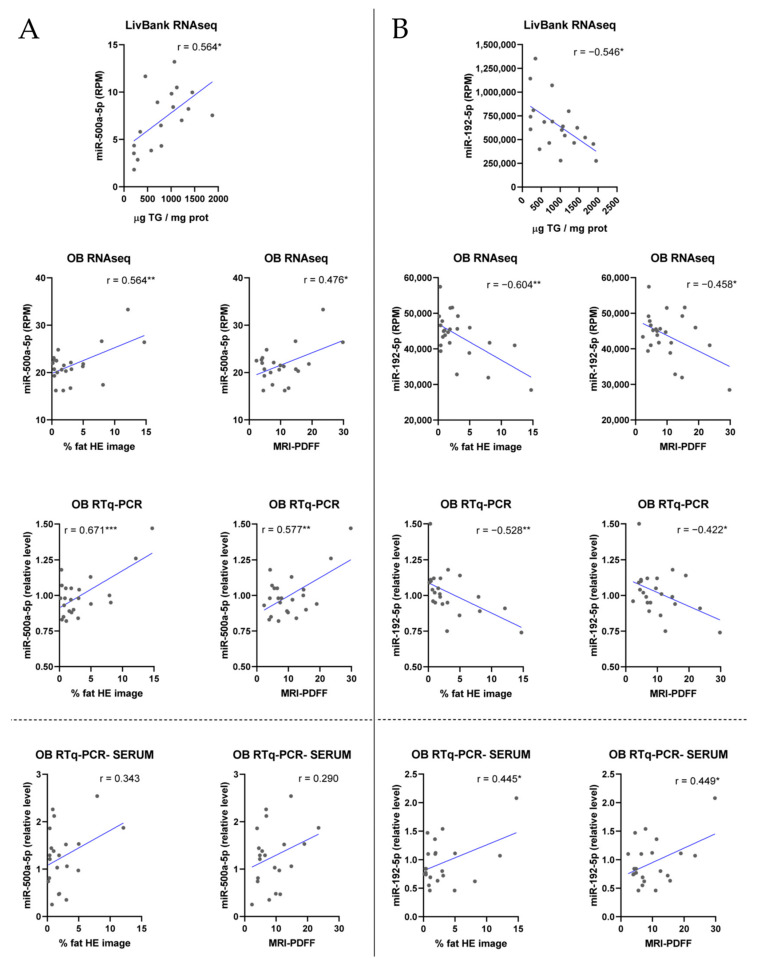
Correlation of representative liver and serum miRNAs with steatosis. Correlation analysis of miR-500a-5p (**A**) and miR-192-5p (**B**) levels in liver (**above** dotted line) and serum (**below** dotted line) with steatosis assessed by TG content (liver biobank) or by liver HE-stained biopsy WSI and MRI-PDFF analyses (morbidly obese patients (OB)). *, *p* < 0.05; **, *p* < 0.01; ***, *p* < 0.001.

The correlation trend (positive or negative) was the same in liver and serum for some miRNAs (e.g., 34a-5p or 500a-5p, Figure 2A). However, other miRNAs showed, in serum, a trend opposite to that in liver (e.g., 192-5p, 145-3p, or 143-3p, Figure 2B). This could be related to different mechanisms by which liver lipids influence miRNAs: they can alter miRNA biogenesis, change the rate of miRNA export, or both.

### 3.4. Development and Validation of a Predictive Model of the Steatosis Percentage Based on Serum miRNAs Correlating with the Hepatic Lipid Content

**Figure 3:**
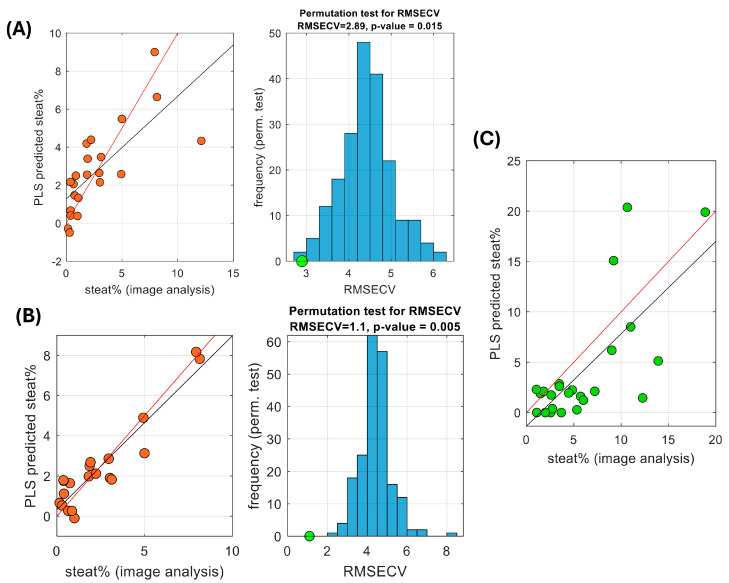
PLS regression, model selection, and validation. Predictive modelling of liver steatosis using PLS regression and assessment of the statistical significance of the CV error by permutation testing in the initial model with 10 miRNAs (**A**) and after 5 feature selection (**B**). (**C**) Results predicted in an external validation set using the PLS model built after 5 feature selection.

A predictive PLS model was built using the complete serum dataset: 10 miRNAs in 24 serum samples (from BS obese patients) vs. the % of liver fat in biopsy digital images, providing a statistically significant RMSECV of 2.89 (permutation test *p*-value = 0.015, Figure 3A).

However, the elimination of features with low predictive performance might provide more stable PLS models, lower prediction errors, and better interpretability than the initial model. Thus, all the possible 252 combinations of five miRNAs were tested and found that the PLS model built from the five miRNAs: 145-3p, 122-5p, 143-3p, 500a-5p, and 182-5p provided the lowest RMSECV = 1.1 (permutation test *p*-value = 0.005, Figure 3B).

Similar PLS models were also developed from the serum miRNA dataset vs. the % of liver fat assessed by MRI-PDFF. When the complete 10 miRNA set was used, the resulting model provided a statistically significant RMSECV of 4.14 (permutation test *p*-value = 0.005). When combinations of five miRNAs were tested, the PLS model built from the combination: 145-3p, 143-3p, 192-5p, 32-5p, and 182-5p provided the lowest RMSECV = 2.59 (permutation test *p*-value = 0.005).

Although LOO-CV demonstrated the validity of the developed model, validation with an external set of samples gives more confidence and reliability to the predictive model.

For the external validation we combined two sets of samples: 15 sera from different morbidly obese BS patients and 10 sera from non-morbidly obese MASLD patients (see Table 1B,C). The only shortcoming was that, for these 10 MASLD patients, liver steatosis was only quantified by biopsy WSI analysis, and MRI-PDFF data was not available. Thus, only biopsy digital image-based models could be applied.

After analyzing the expression of the selected five miRNAs, the PLS model predicting the % of steatosis in liver biopsies showed a good correlation between real and predicted values with a root mean squared error of prediction (RMSEP) of 5.3 (Figure 3C).

Both RMSECV and RMSEP estimate the predictive performance of the model on the model development and validation sets, respectively. An RMSEP of 5.3 means that the model predictions deviate from the true values by 5.3 units on average. The adequacy of this accuracy depends on the context of application, such as the range of steatosis in the population (e.g., <5% to >60%) and the clinical expectations. Furthermore, it is common that RMSEP > RMSECV because RMSECV, estimated from the model development set, does not capture variations in the external validation set, often leading to overly optimistic performance estimates. However, in this study, the RMSEP value is not much higher than the RMSECV, indicating a good generalization of the model.

## 4. Discussion

The approach followed in this study is novel and unique as we only prioritized miRNAs that, as follows:

1. Were not pre-selected; rather, they were identified by high-throughput screening (miRNAseq) in the whole liver miRNome.
2. Showed correlation with the % of steatosis in the liver of two independent cohorts of patients (with biopsy proven steatosis).
3. Showed correlation with the % of steatosis in patients as determined by different alternative linear methods (biopsy WSI analysis, MRI-PDFF, µgTG/mg protein).
4. Demonstrated this correlation using two alternative miRNA expression methods (miRNAseq and RT-qPCR).
5. Showed correlation/association in paired liver and serum samples of the same patients.

A workflow of the whole process is depicted in Supplementary Figure S1.

Among the different, non-invasive image techniques for liver steatosis in MASLD, ultrasonography is probably the most accessible and could be the first-line diagnostic tool for assessing suspected hepatic steatosis or a screening tool for asymptomatic steatosis [18]. However, while ultrasonography could be reliable at detecting moderate steatosis, its sensitivity is poor when <20–30% of hepatocytes are steatotic [18]. For instance, in a comparative study of different imaging techniques, ultrasonography showed the lowest sensitivity (65%) and specificity (77%) in detecting ≥5% histologically defined hepatic steatosis [19]. In addition, ultrasonography usually reports steatosis on a semiquantitative scale (normal, mild, moderate, and severe). Thus, the complementation of widely available imaging techniques with circulating biomarkers could increase accuracy in steatosis diagnosis, and with a quantitative score. The predictive model developed in this study, based on the serum level of five miRNAs, is a non-invasive and cost-effective method that can be easily implemented to screen liver steatosis in large populations. Those patients with a suggestive % of steatosis could be confirmed by image methods.

A key feature in making serum miRNAs excellent potential biomarkers is their stability. They are resistant to different external insults because they do not circulate as free RNA; instead, they are encapsulated in membranous vesicles (micro-vesicles, exosomes, apoptotic bodies), complexed to RNA-binding proteins (e.g., Ago2) or associated with lipoproteins, which protect them from endogenous RNases. Another aspect making miRNA molecules attractive biomarkers is that the RT-qPCR technique used for their detection is extremely sensitive and cost effective [20].

The miRNAseq results disclosed a substantial number of miRNAs (between 102 and 145) with a potential correlation with the % of liver steatosis. However, only a small fraction of them (~25%) were coincident in the two different cohorts of patients. Results suggest that many of the previous associations reported are strongly influenced not only by steatosis but also by cohort specific (confounding) factors. Therefore, many of the miRNAs postulated as predictive MASLD biomarkers in previous studies could have applications only in the specific cohort of patients in which they were discovered, but in order to postulate general miRNA biomarkers, more than one cohort of patients should be investigated.

In the present study, another important advantage was to have paired liver and serum samples from the same patients. In our previous pilot study, different cohorts of patients were used for liver and serum samples [10]. That study showed the feasibility of using miRNAs as quantitative biomarkers to predict the % of steatosis, but only some of the proposed miRNAs in that study have been confirmed in the present study (e.g., miR-98, miR-32, and miR-145). This reinforces the notion that results in different cohorts of patients may be influenced by multiple intrinsic factors in addition to the % of steatosis.

Another important bias in previous studies is that MASLD diagnosis is not biopsy-proven, e.g., [21], thus opening the possibility that false positive and negative patients could be inadvertently included. Our study included not only biopsy-proven patients but also patients with an accurate quantification of steatosis either by biochemical methods (µg TG/mg liver protein), by image methods (MRI-PDFF), or by biopsy WSI analysis (based on the % of the area occupied by lipid droplets in the HE-stained liver sections).

However, our study also has limitations. We are aware that several tissues, and not only the liver, may contribute to serum miRNA levels. Nevertheless, indirect evidence suggests that the liver may be one of the main contributors to the changes observed in the selected serum miRNAs: (a) some of these miRNAs are liver-specific or liver-enriched (e.g., miR-122 or miR-192); (b) for ubiquitously distributed miRNAs, the liver, being one of the largest organs, may contribute significantly; (c) of the 14 miRNAs measured in serum, four did not correlate with steatosis, whereas 10 did as in the liver. It is likely that these 10 miRNAs have the liver as a major contributor; and (d) most of the selected miRNAs had higher expression in the liver (lower Cts) than in the serum (higher Cts). Only for 93-5p, 32-5p, and 182-5p, liver Cts were similar to serum Cts, indicating that, for these miRNAs, other tissues may likely contribute to baseline serum levels.

Regarding the biological relevance of the selected miRNAs that correlate with liver steatosis, most of them appear to be involved in lipid metabolism and homeostasis. The alteration of miRNA expression levels observed in patients with steatosis could be attributed to causal or adaptive mechanisms.

In HepG2 cells, a liver cell-line relevant for HDL biogenesis, miR-145 was found to repress ABCA1 expression and cholesterol efflux [22]. Overexpression of miR-145 in mammary cells increased transcription of genes associated with milk fat synthesis resulting in greater fat droplet formation and TG accumulation. Moreover, in this study, insulin induced gene 1 (INSIG1) was shown to be a direct target of miR-145 [23]. It has also been shown that miR-145 targets FOXO1 and relieves the inhibitory effect of FOXO1 on *SREBP1*, the master activator of lipid synthesis, subsequently leading to increased lipogenesis [24].

In human hepatocytes, miR-143 repressed ANGPTL8 [25]. *ANGPTL8* is predominantly expressed in the liver. It inhibits lipoprotein lipase, and its expression is lower in dyslipidemic patients. miR-143 mimic promoted adipogenesis by accumulating more triglycerides in adipocytes [26]. Similarly to miR-145, miR-143 overexpression remarkably promoted the production of lipid droplets and increased the level of TGs in mammary cells [27].

We observed that miR-145 and miR-143 decreased in the liver as steatosis increased. As these two miRNAs seem to have a pro-steatotic potential, their decrease could be an adaptive mechanism to counteract steatosis.

Regarding miR-192, several studies suggest it is involved in lipid metabolism and can ameliorate steatosis. miR-192 directly acted on the 3′UTR of SREBP1. Indeed, miR-192 mimic or overexpression of miR-192 improved hepatic steatosis by suppressing SREBP1 [28]. In another study, transfection with miR-192-5p mimic and inhibitor in Huh7 hepatic cells induced dramatic repression and promotion of stearoyl-CoA desaturase (SCD-1) expression, respectively [29]. Thus, miR-192-5p demonstrated a negative regulatory role in lipid synthesis. Similarly, the inhibition of miR-192-5p increased the accumulation of hepatic TGs and aggravated hepatic steatosis in NAFLD mice. Yin Yang 1 was identified as the target gene of miR-192-5p, which regulates TG synthesis by activating fatty-acid synthase (FASN) [30].

There are also many reports showing a link between miR-122 and lipid metabolism. Several of them demonstrate that inhibition of miR-122 increases lipid synthesis in the liver [31,32]. Furthermore, the silencing of miR-122 was shown to be an early event in the development of nonalcoholic steatohepatitis both in human patients and in a mouse model [33,34].

In the present study, we found that both miR-192-5p and miR-122-5p were reduced along with increased steatosis in human livers from different cohorts of patients, and according with the above referenced studies, this suggests a causal mechanism between reduced expression of these two miRNAs and more steatosis and MASLD progression.

In an opposite way, miR-34a inhibition suppressed lipid accumulation and improved the degree of steatosis [35]. In another study, overexpression of miR-34a resulted in increased TG accumulation as well as in decreased mitochondrial membrane potential and SIRT1 levels [36]. Thus, miR-34a, contrary to miR-192 and miR-122, seems to play a pro-steatotic role. Our results demonstrate increased miR-34a along with steatosis, which agrees with previous studies suggesting circulating miR-34a as a promising biomarker for MASLD. miR-34a showed elevated serum levels in patients with MASLD and even higher levels in patients with more severe MASH. Moreover, it correlated with steatosis [37,38].

Other miRNAs showing correlation with steatosis, such as miR-32, mimR-98, and miR-182, have also been associated with liver lipid metabolism. miR-32 was shown to directly target INSIG1, which leads to the activation of SREBP and associated lipogenic gene programs, thereby promoting hepatic lipid accumulation and metabolic disorders [39]. The overexpression of miR-98-5p decreased the transcript levels of both gluconeogenic and lipogenic genes, thus reducing hepatic glucose production and fat accumulation in HepG2 cells [40]. miR-182-5p overexpression also decreased lipid accumulation in HepG2 cells following oleic acid administration [41]. Regarding miR-93 and miR-500a, we have not found studies showing a link between these miRNAs and liver lipid metabolism and/or MASLD.

To further investigate the functional relevance of miRNAs associated with hepatic steatosis, we performed in silico analyses to identify miRNA target genes and the connected pathways. To this end, we used the online miRSystem tool, which integrates several miRNA target gene prediction programs (DIANA, miRanda, TargetScan, etc.), validated data on interactions between miRNAs and their target genes (TarBase, miRecords, etc.), and pathway databases [42]. In silico results demonstrate that the miRNAs predicting steatosis potentially target different pathways related to lipid and glucose metabolism, including nuclear receptors (ESR1, HNF4A, PXR, GR, LRH-1, PPARA, THRA, etc.), ChREBP pathways (MLX, PRKACB, PPP2CB, etc.), PPARα pathways (PPARA, PGC1A, ACOX1, ACSL1, ABCA1, TNFRSF21, NFKBIA, etc.), insulin signaling pathways (INSR, IRS2, PIK3CG, AKT3, TSC1, PPP1CC, GYS1, FOXO1, etc.), fatty acid, TG and glycerophospholipid metabolism (ACSL4/1, ELOVL4, MGLL, AGPAT1, BAAT, CROT, GK, LPIN1, ACOX1, CHKA, DGKI, ETNK1, LPGAT1, MBOAT2, etc.), and glucose and pyruvate metabolism, TCA cycle and oxidative phosphorylation (ALDOA, PC, PFKB3/4, GYG2, GYS1, PGK1, PGM1, CS, IDH1, LDHA, PDPR, SDHC, ATP6V1A, COX8C, NDUFC2) (Table S2). Results demonstrated that most of the miRNA selected as biomarkers of steatosis may be involved in pathways affecting steatosis (Table S2), which reinforces previous experimental evidence, as well as the reliability on these circulating biomarkers.

## 5. Conclusions

We have identified a small group of liver miRNAs that correlate with the liver lipid content in MASLD patients from different cohorts. Ten of these liver miRNAs were also detected in the serum of the same patients, where they preserved correlation with liver fat content. Most of these miRNAs appear to be involved in lipid metabolism and may have a casual or adaptive role in MASLD. After the development of a quantitative validated model, we conclude it is possible to predict the percentage of liver steatosis with a low error rate by quantifying five serum miRNAs by an easily implementable and cost-effective RT-qPCR method.

## Figures and Tables

**Table 1:** (**A**). Baseline characteristics of the study cohort for model development: obese BS patients (*n* = 24). (**B**). Baseline characteristics of the model validation cohort: obese BS patients (*n* = 15). (**C**). Baseline characteristics of the model validation cohort: non-morbidly obese MASLD patients (*n* = 10).

(A)											
	Mean	SD	Median	IQR	Min	Max	Disease Stage (% of Patients)				
							**0**	**1**	**2**	**3**	**4**
Age (years)	46	12	47	21	26	63					
Gender Female (%)	71%										
BMI Max (kg/m^2^)	46	8	44	6	36	77					
BMI Surgery (kg/m^2^)	38	5	37	4	31	51					
Glucose (mg/dL)	102	22	92	18	83	172					
HbA1c (%)	5.8	0.8	5.5	0.6	4.7	7.9					
Insulin (µU/mL)	14.7	5.1	13.9	9.8	8.5	24.5					
HOMA-IR	3.7	1.5	3.7	2.0	1.9	7.3					
Cholesterol (mg/dL)	171	51	169	59	70	280					
HDL (mg/dL)	42	11	43	12	26	65					
LDL (mg/dL)	101	48	104	44	10	199					
Triglycerides (mg/dL)	140	62	134	61	67	304					
AST (IU/L)	19	6	17	6	11	38					
ALT (IU/L)	24	13	19	13	10	57					
ALP (IU/L)	79	22	70	24	45	138					
Bilirubin (mg/dL)	0.59	0.61	0.45	0.17	0.25	2.48					
Albumin (g/dL)	4.3	0.3	4.3	0.4	3.6	4.8					
PT (s)	11.5	0.8	11.5	1.1	10.0	12.8					
Hemoglobin (g/dL)	14	2	14	2	12	17					
Platelets (10^3^/µL)	259	57	248	61	174	377					
**Histopathology**											
% Steatosis—Digital HE-Stained Image	3.2	3.9	1.8	2.9	0.2	14.7					
Steatosis	1.1	0.8	1.0	1.0	0	3	21%	50%	25%	4%	
Balooning	0.5	0.7	0.0	1.0	0	2	61%	26%	13%		
Lobular Inflammation	0.7	0.6	1.0	1.0	0	2	35%	61%	4%		
Overall Activity	1.3	1.1	1.0	2.0	0	4	29%	33%	25%	8%	4%
Fibrosis	0.4	0.9	0.0	0.3	0	4	75%	21%	0%	0%	4%
**MRI-PDFF**											
% Steatosis	10	7	8	8	2	30					
**(B)**											
	**Mean**	**SD**	**Median**	**IQR**	**Min**	**Max**	**Disease Stage (% of Patients)**				
							**0**	**1**	**2**	**3**	**4**
Age (years)	51	10	53	14	31	63					
Gender Female (%)	67%										
BMI Max (kg/m^2^)	47	8	46	7	39	73					
BMI Surgery (kg/m^2^)	41	7	38	5	33	63					
Glucose (mg/dL)	96	8	98	14	83	108					
HbA1c (%)	5.3	0.4	5.4	0.5	4.6	6.2					
Insulin (µU/mL)	16.9	6.9	15.9	8.4	6.8	31.8					
HOMA-IR	4.0	1.5	4.2	2.3	1.7	7.3					
Cholesterol (mg/dL)	167	34	166	28	126	271					
HDL (mg/dL)	43	7	42	12	31	53					
LDL (mg/dL)	94	28	90	16	54	177					
Triglycerides (mg/dL)	149	63	148	101	65	274					
AST (IU/L)	20	4	21	6	13	27					
ALT (IU/L)	24	9	23	13	10	40					
ALP (IU/L)	70	13	69	14	50	101					
Bilirubin (mg/dL)	0.76	0.22	0.76	0.29	0.50	1.20					
Albumin (g/dL)	4.4	0.2	4.4	0.2	4.0	4.8					
PT (s)	11.2	0.5	11.1	0.6	10.0	11.9					
Hemoglobin (g/dL)	14	1	14	2	12	17					
Platelets (10^3^/µL)	217	56	211	48	112	307					
**Histopathology**											
% Steatosis—Digital HE-Stained Image	4.7	3.0	3.7	3.0	1.1	12.3					
Steatosis	1.3	0.5	1.0	1.0	1	2	0%	67%	33%	0%	
Balooning	0.7	0.6	1.0	1.0	0	2	40%	53%	7%	0%	
Lobular Inflammation	0.9	0.3	1.0	0.0	0	1	7%	93%	0%	0%	
Overall Activity	1.5	0.8	2.0	1.0	0	3	13%	27%	53%	7%	0%
Fibrosis	0.4	0.5	0.0	1.0	0	1	60%	40%	0%	0%	0%
**MRI-PDFF**											
% Steatosis	12	5	10	6	6	26					
**(C)**											
	**Mean**	**SD**	**Median**	**IQR**	**Min**	**Max**	**Disease Stage (% of Patients)**				
							**0**	**1**	**2**	**3**	**4**
Age (years)	54	10	52	11	42	74					
Gender Female (%)	40%										
BMI (kg/m^2^)	31	4	30	4	25	38					
Glucose (mg/dL)	119	48	97	37	85	241					
HbA1c (%)	6.0	1.3	5.7	0.3	4.6	9.1					
Insulin (µU/mL)	22.2	16.9	15.6	14.2	4.0	61.0					
HOMA-IR	6.4	5.8	4.4	4.6	1.3	17.9					
Cholesterol (mg/dL)	196	31	197	50	149	244					
HDL (mg/dL)	49	24	40	11	26	109					
LDL (mg/dL)	106	32	102	21	73	177					
Triglycerides (mg/dL)	215	128	173	121	91	505					
AST (IU/L)	57	45	36	51	19	156					
ALT (IU/L)	66	41	59	42	19	134					
ALP (IU/L)	72	30	64	46	44	124					
Bilirubin (mg/dL)	0.67	0.29	0.61	0.21	0.25	1.30					
Albumin (g/dL)	4.6	0.2	4.6	0.3	4.4	5.0					
PT (s)	13.8	1.6	14.5	2.9	12.0	16.1					
Hemoglobin (g/dL)	15	1	15	1	12	17					
Platelets (10^3^/µL)	248	48	267	77	173	294					
**Histopathology**											
% Steatosis—Digital HE-Stained Image	7.5	6.2	7.4	9.2	1.1	18.9					
Steatosis	1.7	1.2	2.0	1.8	0	3	20%	20%	30%	30%	
Balooning	0.7	0.7	1.0	1.0	0	2	40%	50%	10%		
Lobular Inflammation	0.8	0.4	1.0	0.0	0	1	20%	80%	0%		
Overall Activity	1.5	1.0	2.0	1.0	0	3	20%	20%	50%	10%	0%
Fibrosis	1.1	1.2	1.0	1.8	0	3	40%	30%	10%	20%	0%
**MRI-PDFF**											
% Steatosis	ND	ND	ND	ND	ND	ND					

**Table 2:** Analysis of correlation of 23 liver miRNAs with steatosis, as assessed by RT-qPCR.

		Obese BS Patients % Fat Biopsy Digital Image		Obese BS Patients MRI-PDFF Dixon		
	miRNA	Pearson r	*p*-Value	Pearson r	*p*-Value	
1	1180-3p	0.28	0.186	0.15	0.493	non-validated
2	1247-5p	0.07	0.755	0.04	0.867	non-validated
3	143-3p	−0.41	0.051	−0.41	0.053	
4	145-3p	−0.48	0.024	−0.51	0.016	
5	145-5p	−0.42	0.049	−0.48	0.025	
6	148b-5p	0.49	0.018	0.38	0.073	
7	15b-3p	0.48	0.021	0.33	0.119	
8	182-5p	0.42	0.048	0.33	0.125	
9	192-5p	−0.53	0.010	−0.42	0.045	
10	194-3p	−0.41	0.045	−0.37	0.078	
11	24-2-5p	−0.31	0.158	−0.52	0.012	
12	30a-5p	−0.11	0.614	−0.06	0.777	non-validated
13	30c-2-3p	0.44	0.038	0.26	0.223	
14	32-5p	−0.47	0.020	−0.50	0.013	
15	34a-5p	0.54	0.006	0.63	0.001	
16	362-3p	0.50	0.015	0.40	0.061	
17	500a-5p	0.67	0.001	0.58	0.004	
18	501-3p	−0.32	0.138	−0.52	0.011	
19	93-5p	0.70	0.000	0.59	0.003	
20	98-5p	0.54	0.009	0.40	0.063	
21	let-7a-3p	0.25	0.231	0.18	0.394	non-validated
22	let-7b-5p	0.39	0.065	0.32	0.132	
23	122-5p	−0.55	0.008	−0.46	0.033	

**Table 3:** Analysis of correlation of 14 serum miRNAs with liver steatosis, as assessed by RT-qPCR.

		Obese BS Patient % Fat Biopsy Digital Image		Obese BS Patient MRI-PDFF Dixon		
	miRNA	Pearson r	*p*-Value	Pearson r	*p*-Value	
1	143-3p	0.51	0.016	0.43	0.045	
2	145-3p	0.66	0.001	0.55	0.010	
3	145-5p	0.01	0.963	−0.01	0.963	non-validated
4	148b-5p	−0.15	0.507	−0.32	0.143	non-validated
5	182-5p	−0.32	0.156	−0.31	0.172	
6	192-5p	0.45	0.038	0.45	0.036	
7	32-5p	0.06	0.793	0.39	0.067	
8	34a-5p	0.34	0.118	0.28	0.201	
9	362-3p	−0.05	0.813	−0.01	0.970	non-validated
10	500a-5p	0.34	0.128	0.29	0.198	
11	501-3p	0.02	0.913	0.01	0.981	non-validated
12	93-5p	−0.34	0.119	−0.36	0.098	
13	98-5p	0.07	0.755	0.45	0.034	
14	122-5p	0.54	0.092	0.22	0.328	

## Data Availability

Sequencing results were deposited in ENA under accession PRJEB53387 and in BioStudies under the accession S-ONTX26. Liver and serum RT-qPCR datasets were deposited in BioStudies under accession S-ONTX27 and S-ONTX28, respectively.

## Supplementary Materials

The following supporting information can be downloaded at https://www.mdpi.com/article/10.3390/biom14111423/s1; Table S1: Correlation between liver miRNA levels, assessed by RNAseq, and the magnitude of liver steatosis by different methods; Table S2: Potential involvement of miRNAs correlating with steatosis in metabolic pathways related to lipid and glucose metabolism; Figure S1: Overview of the study design and results of the identification and confirmation of hepatic microRNAs that correlate with hepatic steatosis.
