# Supplementary material for: The Development of a Non-Invasive Screening Method Based on Serum microRNAs to Quantify the Percentage of Liver Steatosis
Source: Biomolecules. 2024 Nov 8;14(11):1423. doi: 10.3390/biom14111423 (PMC11592063; doi:10.3390/biom14111423)
Supplement: Supplementary file 1 [file biomolecules-14-01423-s001.zip › Figure S1.pdf]

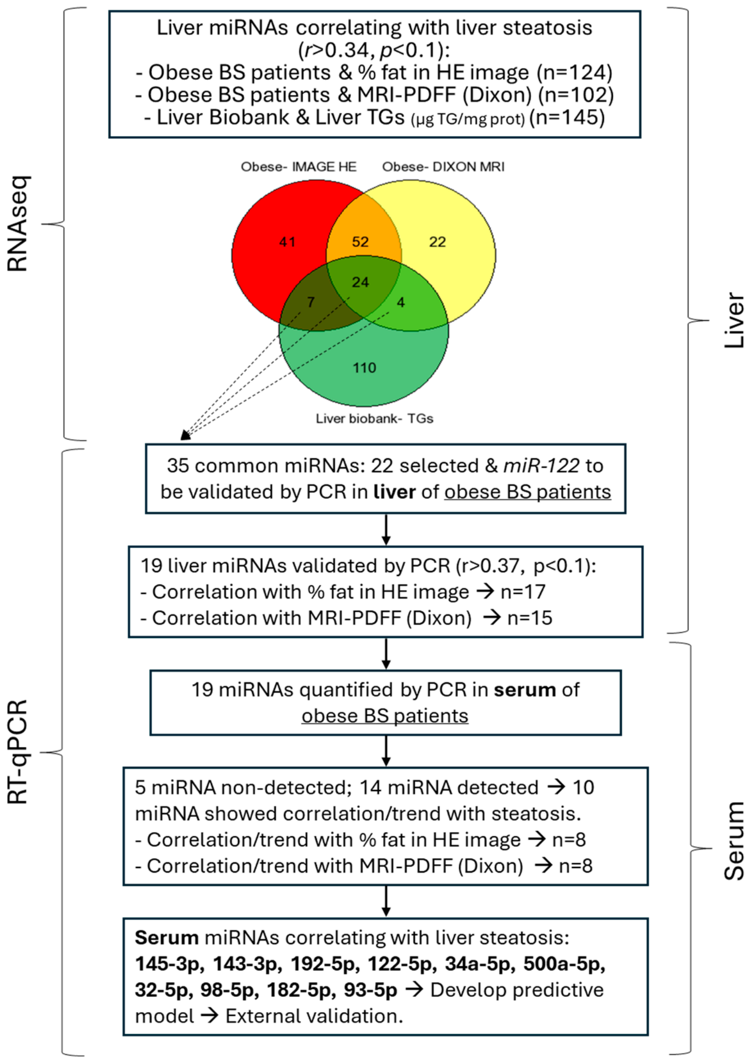

Supplementary Figure S1. **Overview of the study design and results of the identification and confirmation of hepatic microRNAs that correlate with hepatic steatosis**. The study included miRNAseq and RT-qPCR analyses. miRNAseq identified 35 common miRNAs from liver samples correlating with liver steatosis in MASLD patients across three datasets (IMAGE HE, DIXON MRI, and Liver Biobank-TGs). Among them, 23 miRNAs, including miR-122, were also analyzed by RT-qPCR in liver samples from obese BS patients, and confirmed 19 with significant correlations with liver steatosis (17 with digital biopsy images and 15 with MRI-PDFF). Fourteen of these 19 miRNAs were also detected and quantified in serum samples of the same obese BS patients. This analysis identified 10 miRNAs showing consistently associations with liver steatosis across both liver and serum samples, leading to the development of a predictive model. Key serum miRNAs correlating with liver steatosis include: 145-3p, 148-3p, 192-5p, 122-5p, 34a-5p, 500a-5p, 32-5p, 98-5p, 182-5p, 93-5p.
